# A parametric logistic equation with light flux and medium concentration for cultivation planning of microalgae

**DOI:** 10.1098/rsif.2022.0166

**Published:** 2022-06-15

**Authors:** Kazuki Kambe, Yasutaka Hirokawa, Asuka Koshi, Yutaka Hori

**Affiliations:** ^1^ Department of Applied Physics and Physico-Informatics, Keio University, 3-14-1, Yokohama, Kanagawa 223-8522, Japan; ^2^ meravi Inc., 6-11-40, Soka, Saitama 340-0002, Japan

**Keywords:** logistic equation, growth profile, microalgae, cultivation planning, light flux, medium concentration

## Abstract

Microalgae are considered to be promising producers of bioactive chemicals, feeds and fuels from carbon dioxide by photosynthesis. Thus, the prediction of microalgal growth profiles is important for the planning of cost-effective and sustainable cultivation–harvest cycles. This paper proposes a mathematical model capable of predicting the effect of light flux into culture and medium concentration on the growth profiles of microalgae by incorporating these growth-limiting factors into a logistic equation. The specific form of the equation is derived based on the experimentally measured growth profiles of *Monoraphidium* sp., a microalgal strain isolated by the authors, under 16 conditions consisting of combinations of incident light fluxes into culture and initial medium concentrations. Using a cross-validation method, it is shown that the proposed model has the ability to predict necessary incident light flux into culture and initial medium concentration for harvesting target biomass at a target time. Finally, model-guided cultivation planning is performed and is evaluated by comparing the result with experimental data.

## 1. Introduction

Photosynthesis is a beneficial reaction that reconverts atmospheric carbon dioxide produced by the consumption of fossil resources into organic carbon. It is one of the desirable solutions for a sustainable future to substitute photosynthetic products for fossil resources. Microalgae, which grow faster and show higher carbon fixation rates than higher plants, are excellent candidates for carbon neutral producers. Some species of microalgae have been used as live feed for fish larvae and are expected as alternative feedstocks for livestock [1] and aquaculture [2] because of their high nutritional quality. Other species of microalgae were reported as superior producers of bioactive chemicals [3], biofuels [4,5] and biohydrogen [6], which would potentially revolutionize the production of cosmetics, health foods and fossil-based energy. Among them, *Monoraphidium* species classified in Selenastraceae are oleaginous microalgae showing high lipid contents and were reported as promising hosts for biofuel production [5,7–10]. Moreover, recent studies showed that *Monoraphidium* could grow robustly even in wastewater, suggesting that *Monoraphidium* cultivation could become a simultaneous solution for bioremediation and biorefinery [11,12]. To date, optimization of microalgal cultivation has been studied by computational and experimental approaches [13–15]. In these types of optimization, it is a common procedure to optimize the titre of microalgae in a single culture harvest. However, a major challenge in practical algal cultivation lies in the discovery of cultivation conditions that enable continuously stable and cost-effective production over multiple cultivation–harvest cycles. Thus, a model-guided approach to the design of a cultivation plan for long-term cultivation is desirable. In particular, development of a mathematical model capable of predicting the number of cells and harvest time in response to various cultivation conditions will be a key to finding the conditions for sustainable cultivation.

Many existing mathematical models predict the growth rate in response to various environmental factors during cultivation [16,17]. Examples include the Monod growth model describing the effect of specific nutrients [18] and the models of temperature-dependent cell growth [14,15,19]. The effect of light intensity on the growth profile was also modelled in various ways depending on the state of the cell culture [17]. Although the growth rate is affected by numerous factors including nutrient concentration, light intensity, temperature, carbon dioxide concentration and toxic by-products in the medium [20], these models consider the effect of only a few factors based on the assumption that cell growth is ultimately limited by those factors. In contrast to these models, the logistic equation incorporates the bulk effect of multiple growth-limiting factors such as medium concentration and toxic by-products into a single parameter called the carrying capacity of the environment [21]. Thus, the equation was used to fit the growth profiles of a wide range of microorganisms [22–26]. However, since it does not explicitly consider the counteraction of the biomass production to the change of environmental factors, the original logistic equation cannot directly be used for the exploration of the growth conditions in cultivation planning. To overcome the limitations of these models, a recent study proposed a hybrid logistic–Monod model [27]. This model explicitly takes the medium concentration into the variable while the other self-limiting factors are considered in the logistic-type equation. In particular, this model explicitly captures the dynamic interplay of the biomass production and the medium consumption using ordinary differential equations (ODEs) to enable the exploration of cultivation conditions. This idea of modelling the complex interplay of biomass production and environmental factors can be used to build a more advanced hybrid model that includes other important factors for algal cultivation such as light intensity.

**Figure 1.**
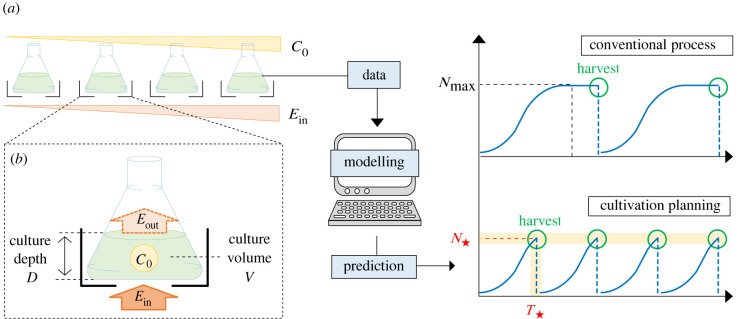
(*a*) Overview of cultivation planning. The symbols (*C*_0_, *E*_in_, *N*_max_, $N_{⋆}$ and $T_{⋆}$) are the initial medium concentration, the incident light flux into culture, the maximum biomass, the target biomass, and the target time, respectively. (*b*) Enlarged view of experimental set-up. The symbol *E*_out_ is the transmitted light flux thorough culture.

This paper proposes an ODE model of the growth profile of microalgae to enable cultivation planning by model-based exploration of the cultivation parameter space, as shown in figure 1*a*. Specifically, the proposed model extends the logistic equation in a way that explicitly incorporates dynamic environmental factors such as light flux being available for a single cell and medium concentration, enabling one to find a cultivation condition that satisfies various constraints of a practical cultivation process such as target biomass and target time. The specific form of the extended logistic equation and its parameters were determined from experimentally measured growth profiles of an originally isolated *Monoraphidium* sp. under different light fluxes into culture and medium concentrations. These experimental data were further used for the cross-validation of the proposed model. The cross-validation result showed that the model could replicate various features of the growth profiles, including the peak biomass and its timing, for different cultivation conditions. Finally, we showcase a procedure of cultivation planning, where the initial medium concentration and the incident light flux into culture are explored to achieve predefined target biomass and target time based on the simulations and some analytic relation of the proposed model.

## 2. Experimental conditions and overview of the proposed model

The goal of cultivation planning is to find parameters of cultivation such as the initial medium concentration *C*_0_ and the incident light flux into culture *E*_in_ that achieve a target biomass $N_{⋆}$ at target time $T_{⋆}$. To this end, we build a mathematical model that can predict the dynamic biomass *N* in response to various cultivation parameters as shown in figure 1*a*. In this section, we first introduce experimental conditions for building the proposed model, and then outline the overview for the proposed model.

### 2.1. Experimental conditions

Microalgae isolated from a freshwater pond in Yoshikawa, Saitama, Japan were named ACCB1808. The 18S ribosomal DNA sequence of ACCB1808 was 99.3%, 99.1% and 99.1% identical to that of *Monoraphidium* sp. LB59, *M. subclavatum* FBCC-A409 and *Monoraphidium* sp. HDMA-11, respectively (electronic supplementary material, figure S1). This result indicated that ACCB1808 belonged to the genus of *Monoraphidium*.

**Figure 2.**
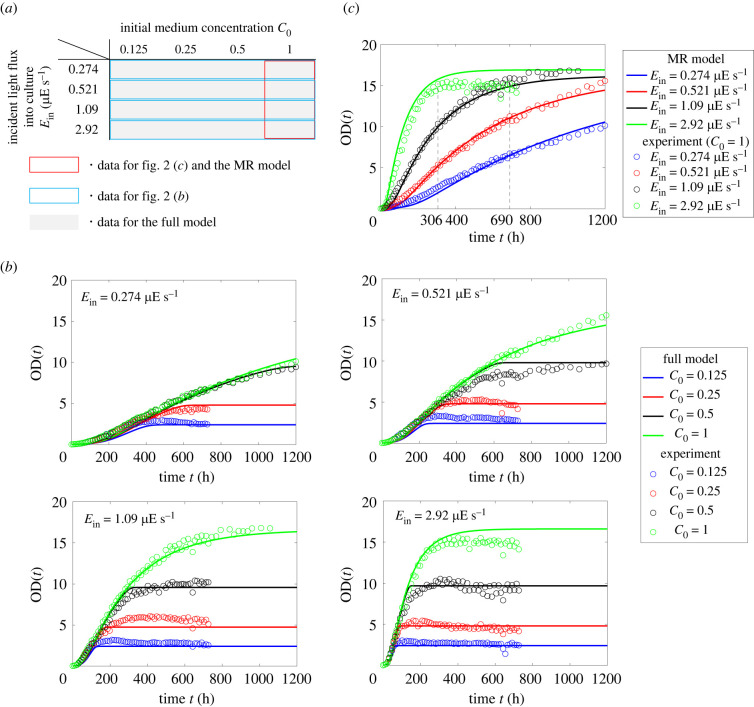
(*a*) Experimental conditions combining initial medium concentrations *C*_0_ and light fluxes into culture *E*_in_. (*b*) Time-series data for different initial medium concentrations *C*_0_ and incident light flux into culture *E*_in_. Circles and solid lines show experimental data and predicted results of the full model described in §4, respectively. (*c*) Time-series data for the four incident light fluxes into culture *E*_in_ with *C*_0_ = 1. Circles and solid lines show experimental data (repeat of the data in (*b*)) and predicted results of the MR model described in §3, respectively.

To measure the growth profile for modelling, ACCB1808 cultures were incubated in 300 ml flasks containing 200 ml of modified BG11 medium. The details of experimental conditions including the medium composition are summarized in appendix A. Flasks containing culture were directly placed onto a white LED light whose intensity measured as photosynthetic photon flux density (PPFD) was 1034 μE m^−2^ s^−1^. To strictly regulate the incident light flux into culture, black drawing paper with a 3 cm radius hole (area of 2.826 × 10^−3^ m^2^) was inserted between the flasks and the LED light. The flasks were placed in an enclosure made with the same drawing paper as shown in figure 1*b*. The incident light intensities to flasks measured as PPFD were adjusted to 1034, 386.7, 184.8 and 96.8 μE m^−2^ s^−1^ by inserting sheets of papers between the flasks and the LED light. Owing to strict regulation of the illuminated area (2.826 × 10^−3^ m^2^), the incident light fluxes into culture *E*_in_ were calculated as 2.92, 1.09, 0.521 and 0.274 μE s^−1^. The concentration of modified BG11 medium *C* was defined as 1, and the initial medium concentrations *C*_0_ were adjusted to 0.5, 0.25 and 0.125 by dilution. Although the medium is composed of different nutrients consumed at various rates upon growth, we here assume that there is a rate-limiting nutrient species and use the single variable *C* to capture the growth-limiting effect by that nutrient. ACCB1808 cultures were incubated under 16 conditions consisting of combinations of four patterns of initial medium concentrations *C*_0_ and four patterns of incident light fluxes into culture *E*_in_ as shown in figure 2*a*. These experimental data were used for building and evaluating the proposed model in the following sections.

### 2.2. Overview of the proposed model

The growth profiles of experimental data shown in figure 2*b* indicated that the growth rate and the biomass accumulation were dependent on the incident light flux into culture *E*_in_ and the initial medium concentration *C*_0_, respectively. In initial growth phase, the biomass *N* increased exponentially. Next, after following a linear increase, the growth rate gradually decreased and levelled off. These phase transitions were possibly caused by photoinhibition, insufficient light absorption at high biomass, depletion of the medium concentration, and increase of inhibitory substances [28,29]. The state of culture used for subculture often affected the initial growth rate in the next cultivation. Specifically, the culture state before stationary phase was preferable for use in subculture. To harvest the culture in the preferable state, it is important to predict not only a target biomass $N_{⋆}$ but also the target time $T_{⋆}$ at which the biomass reaches $N_{⋆}$. Moreover, for practical operation, the target values ($N_{⋆}$ and $T_{⋆}$) have to be decided from various viewpoints such as cost effectiveness and the schedule of operators. A mathematical model would be a desirable tool for exploring cultivation conditions satisfying these target values.

In what follows, we build a mathematical model capable of predicting the growth kinetics of ACCB1808 culture for various cultivation conditions. In particular, we are interested in the growth kinetics in response to light flux into culture *E*_in_ and medium concentration *C* since these parameters largely affect the growth profile as shown in figure 2*b*. Since the increase of biomass decreases available light flux for a single cell even if light flux into culture *E*_in_ is constant during cultivation, available light flux for a single cell is one of the key variables for the prediction of the dynamic biomass change. In this paper, light flux being available for a single cell is called light flux per cell *L*. The effect of light flux per cell on the growth kinetics will be discussed in §3.

We develop an ODE model of the growth kinetics based on the logistic equation [21]

2.1 $$\frac{dN(t)}{dt}=r\left(1-\frac{N(t)}{\Gamma }\right)N(t),$$

where *N*(*t*) is the biomass at time *t*, *r* is the maximum specific growth rate and Γ is the carrying capacity of the environment, or the maximum achievable population size. The first term of the logistic equation represents the growth due to proliferation, and the second term collectively accounts for growth-limiting factors such as toxic by-products and reactive oxygen species.

Unlike the original logistic equation [21], we assume that the maximum specific growth rate *r* is dependent on the light flux per cell *L*, and the medium concentration *C*, that is, *r* := *r*(*L*, *C*). As discussed later in detail, *L* and *C* are subject to dynamic change since these two variables are affected by biomass *N*(*t*). The combined effect of these factors is incorporated into the maximum specific growth rate *r*(*L*, *C*) by

2.2 $$r(L,C)\;:=\mu r_{\mathrm{light}}(L)r_{\mathrm{medium}}(C),$$

where *μ* is a constant, and *r*_light_(*L*) and *r*_medium_(*C*) are functions of light flux per cell and medium concentration. These functions take values between 0 and 1. The specific forms of these functions are defined in §§3 and 4. The functions *r*_light_(*L*) and *r*_medium_(*C*) represent the growth-limiting effect due to insufficient absorption of light flux per cell and insufficient nutrients, respectively. In other words, *r*(*L*, *C*) ≃ *μ* when the light flux per cell *L* and the medium concentration *C* are sufficiently high such that *r*_light_(*L*) ≃ 1, and *r*_medium_(*C*) ≃ 1, while *r*(*L*, *C*) ≃ 0 when *L* or *C* is close to 0.

## 3. Logistic equation with light flux per cell

In the previous section, the maximum specific growth rate *r* is defined by the functions of the medium concentration *C* and of the light flux per cell *L*. When the medium concentration *C* is sufficiently high, the maximum specific growth rate *r* is only dependent on the light flux per cell *L*. In this section, we consider this special case and formulate a logistic equation that incorporates the effect of the light flux per cell *L*, which we call the medium-rich model or the MR model for short.

### 3.1. Modelling of the medium-rich model

We incorporate the effect of the light flux per cell *L* on the growth kinetics by defining

3.1 $$r_{\mathrm{light}}(L)\;:=\frac{L(N(t),E_{\mathrm{in}})}{\lambda_{L}+L(N(t),E_{\mathrm{in}})},$$

where *λ*_*L*_ is a half-velocity constant satisfying *r*_light_(*λ*_*L*_) = 1/2.

The light flux per cell *L*(*N*(*t*), *E*_in_) is dependent on the incident light flux into culture *E*_in_ and the biomass *N*(*t*). The light flux absorbed by the culture is expressed by *E*_in_ − *E*_out_ where *E*_out_ is the transmitted light flux through culture as illustrated in figure 1*b* (see details about *E*_out_ in appendix B). Thus, the absorbed light flux per cell *L*(*N*(*t*), *E*_in_) is written as (*E*_in_ − *E*_out_)/*N*(*t*) by assuming that light flux is uniformly absorbed by cells in culture. Moreover, the attenuation of transmitted light in the culture obeys the Lambert–Beer Law [30], which states the exponential decrease of light flux with the path length of light flux and the concentration of the solution (biomass concentration) (see details in appendix C). Thus, the absorbed light flux per cell *L*(*N*(*t*), *E*_in_) is

3.2 $$L(N(t),E_{\mathrm{in}})=\frac{E_{\mathrm{in}}-E_{\mathrm{out}}}{N(t)}=\frac{1-10^{-K\cdot N(t)/V\cdot D}}{N(t)}E_{\mathrm{in}},$$

where *K* is the cell-specific extinction coefficient, *D* is the culture depth, and *V* is the culture volume as illustrated in figure 1*b*. It should be noted that, in general, the profile of the growth rate functions of light flux per cell could be sigmoidal at low light flux per cell *L* due to the minimum light flux required for cell growth, and be decreasing at high light flux due to photoinhibition [28]. Thus, equation (3.1) is an approximation model that only captures the increasing and the saturation phase of the growth rate in the mild light flux condition, where the cultivation is mainly performed.

When the nutrients in the medium are sufficient and are not rate-limiting factors, *r*_medium_(*C*) = 1 holds. Thus, it follows that

3.3 $$\frac{dN(t)}{dt}=\mu \frac{L(N(t),E_{\mathrm{in}})}{\lambda_{L}+L(N(t),E_{\mathrm{in}})}\left(1-\frac{N(t)}{\Gamma }\right)N(t),$$

by substituting equation (3.1) into equation (2.1). In what follows, equation (3.3) is called the MR model. The MR model allows us to assess some of the parameters of equations (2.1) and (2.2) by using experimental data taken under the conditions with sufficient medium concentration as shown in the next subsection. The other parameters that appear in *r*_medium_(*C*) can then be assessed by subsequent experiments, which will be discussed in §4. The two-step parameter evaluation helps avoid overfitting to a single experimental datum.

### 3.2. Parameter evaluation of the medium-rich model

Experiments were conducted to assess the parameters *K*, *μ*, *λ*_*L*_ and Γ of the MR model (equation (3.3)). Firstly, the extinction coefficient *K* being specific to ACCB1808 was evaluated by the method of Masuda *et al.* [30]. PPFD was measured in ACCB1808 culture with various biomass concentrations and culture depths (see details in appendix C). The relative logarithmic PPFD was negatively correlated with biomass concentrations and culture depths, indicating that the Lambert–Beer Law was obeyed in ACCB1808 culture. When the units of culture depth and biomass concentration were defined as ‘cm’ and ‘cell ml^−1^’, the extinction coefficient *K* was evaluated as *K* = 5.1 × 10^−9^ ml cm^−1^ cell^−1^.

Using this extinction coefficient, we further performed evaluation of *μ*, *λ*_*L*_ and Γ in the MR model (equation (3.3)) based on the growth profile of ACCB1808 culture (see details about cultivation conditions in appendix A).

Experiments were conducted under a total of 16 different conditions as shown in figure 2*a*, where the growth curves were obtained for combinations of four different light fluxes into culture, 0.274, 0.521, 1.09 and 2.92 μE s^−1^, and four initial medium concentrations, 0.125, 0.25, 0.5 and 1. Optical density at 730 nm (OD) was measured as a proxy of biomass *N*(*t*) (see details in appendix C).

The parameters were then fitted to the four growth kinetics with the sufficient medium concentration, i.e. *C*_0_ = 1 in figure 2*c*. Specifically, the culture volume *V*, the culture depth *D*, the initial optical density OD_0_ and the evaluated extinction coefficient *K* were set in equation (3.3) as shown in table 1. When 200 ml of culture was put into a 300 ml flask, culture depth corresponded to 3.7 cm. The culture depth *D* and the culture volume *V* were assumed to remain constant during cultivation. For *E*_in_ = 1.09 and 2.92 μE s^−1^, time-series data were fitted only for the first 690 h and 306 h, respectively, since the medium concentration could become a rate-limiting factor, violating the assumption of the MR model, when the culture reached stationary phase.

**Table 1.** Initial values and assessed extinction coefficient *K*.

parameter	unit	value
*V*	ml	200
*D*	cm	3.7
OD_0_	—	0.025
*K*	ml cm^−1^ cell^−1^	5.1 × 10^−9^

The assessed parameters are shown in table 2. This result implied that the maximum specific growth rate *r*(*L*, *C*) ≃ *μr*_light_(*L*) was between 0 and 0.194 h^−1^ depending on the light flux per cell *L* when the medium concentration was high, i.e. *r*_medium_(*C*) ≃ 1. The carrying capacity Γ was evaluated as 99.9 × 10^9^ cell. The biomass concentration 99.9 × 10^9^/200 cell ml^−1^ can be converted into OD of 16.6. This indicated that the value of OD would never exceed 16.6 regardless of the medium concentration.

**Table 2.** List of evaluated parameters of the proposed model.

parameter	unit	assessed value
*μ*	h^−1^	0.194
*λ*_*L*_	μE s^−1^ cell^−1^	1.90 × 10^−6^
Γ	cell	99.9 × 10^9^
*ξ*_*C*_	—	0.012
*α*	cell^−1^	8.7 × 10^−12^

To evaluate the predictive ability of the MR model, the predicted results of the MR model were further compared with the experimental data using leave-one-out cross-validation (LOOCV) [31], where three of the four experimental conditions were grouped together for parameter evaluation and the other was used for prediction. Specifically, the parameters (*μ*, *λ*_*L*_ and Γ) were fitted to the three growth curves with *C*_0_ = 1. Then, the model was used to predict the growth kinetics as shown in figure 2*c*. Figure 2*c* shows that the MR model was capable of predicting the growth kinetics before reaching stationary phase, where the decrease in medium concentration had little effect on cell growth.

In the next section, the MR model is used to build a full model that captures the effect of both light flux per cell *L* and medium concentration *C* on cell growth.

## 4. Logistic equation with light flux per cell and medium concentration

A standing assumption of the MR model is that the medium concentration *C*(*t*) is sufficiently high so that the maximum specific growth rate *r* is independent of *C*(*t*). In this section, we will extend the MR model (equation (3.3)) to remove this assumption and explicitly incorporate the effect of the medium concentration on the maximum specific growth rate.

### 4.1. Modelling of logistic equation with light flux and medium concentration

The experimental data in figure 2*b* suggest that the medium concentration *C*(*t*) does not affect the growth profile just before the stationary phase is reached.

Based on this observation, we incorporate the rate-limiting effect of the medium concentration using the Monod-type model [18]

$$r_{\mathrm{medium}}(C(t))\;:=\frac{C(t)}{\xi_{C}+C(t)},$$

where *ξ*_*C*_ is a half-velocity constant.

We assume that nutrients in the medium are mainly used for cell growth, and the consumption rate is proportional to the growth rate d*N*(*t*)/d*t*. Consequently, the mathematical model that incorporates the effect of both the light flux per cell *L*(*N*(*t*), *E*_in_) and the medium concentration *C*(*t*) is obtained as

4.1a $$\frac{dN(t)}{dt}=\mu \frac{C(t)}{\xi_{C}+C(t)}\frac{L(N(t),E_{\mathrm{in}})}{\lambda_{L}+L(N(t),E_{\mathrm{in}})}\left(1-\frac{N(t)}{\Gamma }\right)N(t)$$

and

4.1b $$\frac{dC(t)}{dt}=-\alpha \frac{dN(t)}{dt},$$

where *α* is a parameter representing consumption of the medium concentration per a unit increase of the biomass, and *L*(*N*(*t*), *E*_in_) is defined by equation (3.2). Equation (4.1) is called the full model in the following sections. Defining the initial biomass *N*(0) by *N*_0_, equation (4.1) can equivalently be expressed as

4.2 $$\frac{dN(t)}{dt}=\mu \frac{C_{0}-\alpha (N(t)-N_{0})}{\xi_{C}+C_{0}-\alpha (N(t)-N_{0})}\frac{1-10^{-K\cdot N(t)/V\cdot D}}{\lambda_{L}\cdot E_{\mathrm{in}}^{-1}\cdot N(t)+1-10^{-K\cdot N(t)/V\cdot D}}\left(1-\frac{N(t)}{\Gamma }\right)N(t),$$

since equation (4.1*b*) implies

4.3 $$C(t)=C_{0}-\alpha (N(t)-N_{0}),$$

and *E*_in_ > 0 after starting cultivation.

It should be noted that the parameters *K*, *μ*, *λ*_*L*_ and Γ are assessed in the MR model as described in §3.2. The other parameters *ξ*_*C*_ and *α* can be assessed using the 16 experimental data for the different incident light fluxes into culture *E*_in_ and the initial medium concentrations *C*_0_ in figure 2*b*.

### 4.2. Parameter assessment in the full model

The parameters *ξ*_*C*_ and *α* in the full model (equation (4.1)) were assessed using the 16 experimental data in figure 2*b*. Specifically, the initial parameters, *V*, *D* and OD_0_, and the assessed extinction coefficient *K* in table 1 were used. The parameters *μ*, *λ*_*L*_ and Γ obtained using the MR model in §3.2 were set (table 2). Then, the least-square solution was searched for *ξ*_*C*_ and *α*. The evaluated parameters are shown in table 2.

The generalizability of the full model was also evaluated by LOOCV [31] using the experimental conditions matrix in figure 2*a*. Specifically, for each combination of the light flux into culture *E*_in_ and the initial medium concentration *C*_0_, the parameters *ξ*_*C*_ and *α* were assessed with the other 15 experimental data. Then, the model was used to predict the growth kinetics as shown in figure 2*b*. The simulated growth kinetics showed agreement with the experimental data in that the dynamics of the growth rate was dependent on the light flux into culture *E*_in_ before reaching stationary phase, while the maximum biomass was dependent on the initial medium concentration *C*_0_. The result of LOOCV in figure 2*b* also suggests that the model can predict the maximum biomass *N*_max_ or its corresponding maximum OD. These results will be more quantitatively evaluated in the next section along with the demonstration of cultivation planning.

## 5. Demonstration of cultivation planning

The goal of cultivation planning is to find the initial medium concentration $C_{0}^{⋆}$ and the incident light flux into culture $E_{\mathrm{in}}^{⋆}$ to achieve a predefined target biomass $N_{⋆}$ at target time $T_{⋆}$. In a typical cultivation process, cells are harvested before reaching the stationary phase to avoid the carry-over of potentially toxic by-products in subculture. Thus, $C_{0}^{⋆}$ and $E_{\mathrm{in}}^{⋆}$ should be planned so that biomass reaches $N_{⋆}$ at $T_{⋆}$.

### 5.1. Prediction of initial medium concentration $C_{0}^{⋆}$ for target biomass $N_{⋆}$

In a typical cultivation cycle, culture is harvested before reaching stationary phase to maintain a preferable culture state in subculture. In other words, target biomass $N_{⋆}$ should be set less than the maximum biomass at stationary phase *N*_max_, e.g. $N_{⋆}=0.8N_{\max}$. Thus, prediction of the maximum biomass *N*_max_ or its corresponding maximum OD in response to cultivation conditions such as incident light flux into culture *E*_in_ and initial medium concentration *C*_0_ is important in cultivation planning. In theory, *N*_max_ is the biomass at steady state, at which d*N*(*t*)/d*t* = 0. The steady state is achieved when either the biomass *N*(*t*) reaches the carrying capacity Γ, i.e. N(t)=Γ, or the medium concentration is depleted, i.e. *C*(*t*) = 0. Thus, *N*_max_ can be analytically calculated from equations (4.1) and (4.3) as

5.1 $$N_{\max}=\min\left(\frac{C_{0}}{\alpha }+N_{0},\Gamma \right).$$

It should be noted that the biomass *N*(*t*) can be converted to OD by

5.2 $$\mathrm{OD}=\frac{N}{V}\frac{1}{30.1\times 10^{6}}$$

and dry weight per OD is 0.213 mg ml^−1^. Equation (5.1) implies that the maximum biomass *N*_max_, or its corresponding maximum OD, is obtained from an initial medium concentration *C*_0_ when $C_{0}<\alpha (\Gamma -N_{0})$. This analytic solution provides a crude estimation of biomass at stationary phase for a given initial medium concentration.

**Figure 3.**
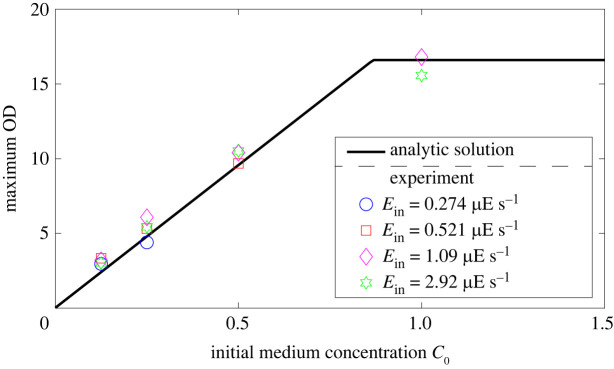
Maximum OD calculated from the analytic solution (equations (5.1) and (5.2)) and measured by experiments in figure 2*b*.

Figure 3 shows the maximum OD predicted from equations (5.1) and (5.2), and measured by the experiments in figure 2*b*, where the parameters *α* and Γ in table 2 and the initial OD_0_ in table 1 corresponding the initial biomass *N*_0_ were used for calculation. The results show that the maximum OD is determined from the initial medium concentrations *C*_0_ and is independent of the incident light fluxes into culture *E*_in_. Thus, a predefined target biomass $N_{⋆}$ or its corresponding target OD for cultivation planning is obtained by simply selecting the initial medium concentration $C_{0}^{⋆}$, which can be calculated from equation (5.1). Once the initial medium concentration $C_{0}^{⋆}$ is fixed, the time when biomass reaches $N_{⋆}$ can be adjusted by incident light flux into culture *E*_in_. In the next subsection, we will give a demonstration to select the incident light flux into culture $E_{\mathrm{in}}^{⋆}$ for target time $T_{⋆}$.

### 5.2. Prediction of incident light flux into culture $E_{\mathrm{in}}^{⋆}$ for target time $T_{⋆}$

Once the initial medium concentration $C_{0}^{⋆}$ is selected based on equation (5.1), the next goal is to seek the incident light flux into culture $E_{\mathrm{in}}^{⋆}$ that achieves target biomass $N_{⋆}$ at target time $T_{⋆}$. The incident light flux into culture $E_{\mathrm{in}}^{⋆}$ is explored by running simulations of the full model (equation (4.1)). Since the target biomass $N_{⋆}$ is often set less than the maximum biomass at stationary phase in practical cultivation, let us suppose, for example, that the target biomass $N_{⋆}$ is around 70–90% of the maximum biomass. Then, the expected harvest time at which the biomass reaches the target biomass can be computed from the growth kinetics simulated by equation (4.1) for each *E*_in_.

**Figure 4.**
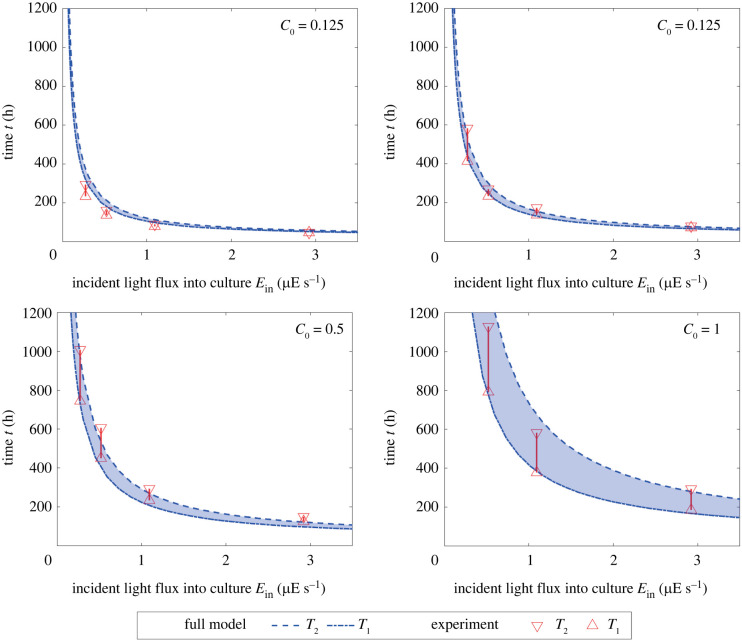
Span of target time obtained by simulations of the full model (equation (4.1)) and measured by experiments in figure 2*b*.

Figure 4 shows the time spans in which the biomass reaches 70–90% of its maximum value for different incident light fluxes into culture *E*_in_. In figure 4, *T*_1_ and *T*_2_ represent the simulated lower and upper bound of harvest time at which biomass reaches 0.7*N*_max_ and 0.9*N*_max_, respectively. The parameters in tables 1 and 2 were used for these simulations of the full model (equation (4.1)). The experimental data in figure 4 are obtained from the time-series data in figure 2*b*.

Figure 4 shows agreement of the computationally predicted time spans with the experimental data. Thus, the incident light flux into culture $E_{\mathrm{in}}^{⋆}$ that enables one to harvest target biomass at target time can be computationally determined based on the full model (equation (4.1)). Thus, combining with the prediction of the initial medium concentration $C_{0}^{⋆}$ in §5.1, one can find cultivation conditions $(C_{0}^{⋆}$ and $E_{\mathrm{in}}^{⋆})$ satisfying the predefined constraints $(N_{⋆}$ and $T_{⋆})$, fulfilling the goal of cultivation planning.

## 6. Conclusion

Microalgae are photosynthetic organisms that have high potential as carbon neutral producers and alternative feedstocks for livestock [1] and aquaculture [2]. Prediction of biomass in cultivation of microalgae is a difficult task due to the complex interplay of growth conditions such as light flux, medium concentration, and temperature [20]. As a result, experimental conditions for harvesting target biomass at target time, i.e. cultivation plans, are often sought empirically by operators.

This paper has proposed an ODE model for predicting the growth profile of microalgae in response to medium concentration and light flux into culture to help operators with cultivation planning. The proposed model has been built by extending the logistic equation in two steps based on the experimentally obtained growth profile of ACCB1808 (*Monoraphidium* sp.) under 16 conditions consisting of the combinations of incident light fluxes into culture and initial medium concentrations. Specifically, we have firstly constructed the MR model (equation (3.3)) that considers only the effect of light flux into culture, assuming that the medium concentration is high. In other words, the MR model captures the growth profile before reaching stationary phase under sufficiently high initial medium concentration conditions. Next, we have extended the MR model to incorporate the effect of the medium concentration (equation (4.1)), where the Monod-type model was introduced based on the experimentally measured growth profile. The predictive ability of the proposed model has then been evaluated by a cross-validation method, and it has been shown that the predicted growth kinetics agrees with experimental data as shown in figure 2*b*. Finally, model-guided cultivation planning has been shown as a demonstration example, where the initial medium concentration and the incident light flux into culture have been planned for harvesting predefined target biomass at target time. The proposed model streamlines the planning process of cultivation cycles that satisfy various practical demands such as cost effectiveness and the schedule of operators.

## Figures and Tables

**Figure 5.**
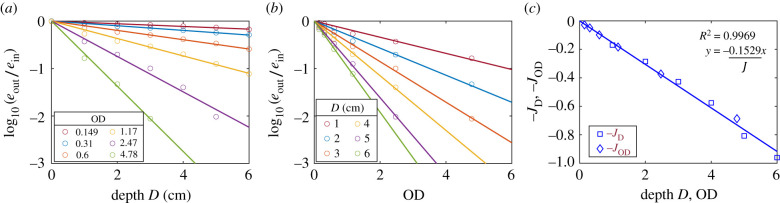
(*a*) Relationships between relative logarithmic PPFD ( =log(*e*_out_/*e*_in_)) and culture depths *D* at particular OD (OD = 0.149–4.78). Circles and solid lines show experimental data and the fitted line with slope (−*J*_OD_), respectively. (*b*) Relationships between relative logarithmic PPFD and ODs at particular culture depth (1–6 cm). Circles and solid lines show experimental data and the fitted line with slope (−*J*_D_), respectively. (*c*) Relation of *J*_OD_ and *J*_D_ with depth *D* and OD. Diamonds and squares show slopes −*J*_OD_ and −*J*_D_ obtained in (*a*) and (*b*), respectively. Solid line shows slope of the fitted line for *J*.

## Data Availability

The program codes used in this study are available at GitHub (https://github.com/hori-group/logistic_eq_for_cultivation_planning). The data are provided in electronic supplementary material [32].

## Appendix A. Experimental conditions for cultivation

All ACCB1808 cultures were incubated in a space of which room temperature was controlled at $25^{\circ }C$ with continuous aeration including 1.5% carbon dioxide. LED light was used for growth, and light intensities were altered for incubation conditions. PPFD in each condition was measured by using a Spectromaster C-7000 (SEKONIC; Tokyo, Japan). Optical density at 730 nm (OD), which is proportional to biomass concentration *N*/*V*, where *N* is the biomass and *V* is the culture volume, was measured by using a V-700 spectrophotometer (JASCO; Tokyo, Japan). Biomass concentration *N*/*V* was evaluated by using a microscope (Olympus; Tokyo, Japan) and a Thoma chamber.

For isolation and preculture, liquid and solid BG11 medium was used containing the following components (per litre): 1.5 g of NaNO_3_, 31.4 mg of K_2_HPO_4_, 73.9 mg of MgSO_4_ · 7H_2_O, 36.8 mg of CaCl_2_ · 2H_2_O, 20.1 mg of Na_2_CO_3_, 1.12 mg of EDTA 2Na, 6.09 mg of citrate, 10.15 mg of ferric ammonium citrate, 1 ml of A6 solution. A6 solution included the following components (per litre): 2.86 g of H_3_BO_4_, 1.81 g of MnCl_2_ · 4H_2_O, 0.22 g of ZnSO_4_ · 7H_2_O, 0.39 g of Na_2_MoO_4_ · 2H_2_O, 0.079 g of CuSO_4_ · 5H_2_O, 0.049 g of Co(NO_3_)_2_ · 6H_2_O. To prepare solid medium, 15 g l^−1^ agar was supplemented.

The growth profiles of ACCB1808 in different concentrations (from 73.9 to 739 mg l^−1^) of MgSO_4_ 7H_2_O in BG11 medium indicated that sixfold concentration (443 mg l^−1^) was appropriate (data not shown). Therefore, MgSO_4_ fortified medium was called modified BG11 medium and used for main culture cultivation.

In preculture cultivation, cells were inoculated into 60 ml of BG11 medium in a 100 ml test tube and incubated under continuous illumination (40 μE m^−2^ s^−1^) and aeration including 1.5% carbon dioxide. Cells of preculture in late logarithmic growth phase were inoculated into 200 ml of modified BG11 medium in a 300 ml flask to an initial optical density at 730 nm (OD_0_) of 0.025.

## Appendix B. Relation between incident light flux and its density

The incident light flux into culture *E*_in_ can be calculated by multiplication of incident light flux density *e*_in_ and illuminated area (2.826 × 10^−3^ m^2^):

$$E_{\mathrm{in}}=e_{\mathrm{in}}\times 2.826\times 10^{-3}.$$

Assuming that the incident light linearly passes culture, the transmitted light flux through culture *E*_out_ is also calculated by

$$E_{\mathrm{out}}=e_{\mathrm{out}}\times 2.826\times 10^{-3}.$$

Based on the Lambert–Beer Law as mentioned in appendix C, the light flux per cell *L*(*N*(*t*), *E*_in_) is obtained as equation (3.2) in §3.1.

## Appendix C. Evaluation of extinction coefficient *K* based on Lambert–Beer Law

The measured PPFD under conditions with different culture depths *D* and optical densities at 730 nm (ODs) are summarized in table 3. It should be noted that the unit of culture depth *D* is centimetre (cm) and that OD is proportional to the biomass concentration *N*/*V*, where *N* is the biomass and *V* is the culture volume. In table 3, *e*_in_ and *e*_out_ correspond to PPFD in culture solution without cells and in culture with various ODs (OD = 0.149–4.78), respectively.

**Table 3.** PPFD for different depth *D* and OD.

	PPFD (μE s^−1^ m^−2^)						
	*e*_in_	*e*_out_					
*D* (cm)	OD = 0	OD = 0.149	OD = 0.310	OD = 0.600	OD = 1.17	OD = 2.47	OD = 4.78
0	834.3	829.0	834.0	824.5	818.0	842.0	839.5
1	419.3	386.5	365.0	300.5	226.5	155.5	69.0
2	203.3	175.0	157.0	120.0	77.0	40.0	9.5
3	114.3	95.0	82.0	58.0	33.5	11.5	1.0
4	75.0	58.5	48.0	31.0	15.0	3.0	N/A
5	52.0	38.0	29.5	17.5	6.5	0.5	N/A
6	39.0	26.0	20.0	10.0	3.0	N/A	N/A

OD can be converted to biomass concentration *N*/*V* by

C 1 $$\frac{N}{V}=\mathrm{OD}\times 30.1\times 10^{6}.$$

Thus, ODs of 0.149, 0.310, 0.600, 1.17, 2.47 and 4.78 in table 3 correspond to 4.55 × 10^6^, 10.1 × 10^6^, 17.6 × 10^6^, 34.9 × 10^6^, 69.8 × 10^6^ and 144 × 10^6^ cell ml^−1^, respectively.

Figure 5*a*,*b* shows relative logarithmic PPFD ( =log(*e*_out_/*e*_in_)) for various culture depths *D* at particular OD (OD = 0.149–4.78) and for various ODs at particular culture depth (1–6 cm), respectively. The relative logarithmic PPFD was negatively proportional to culture depth *D* and OD. This suggested that the Lambert–Beer Law was applicable to the culture solution. In other words,

C 2 $$\log_{10}\left(\frac{e_{\mathrm{out}}}{e_{\mathrm{in}}}\right)=-K\cdot \frac{N}{V}\cdot D=-J\cdot \mathrm{OD}\cdot D,$$

where *K* and *J* are the extinction coefficients defined for biomass concentration *N*/*V* and OD, respectively. The relationship between *K* and *J* can be written by *J* = *K* × 30.1 × 10^6^, according to equation (C1). It should be noted that, by convention, the natural number e is also used as the base of logarithm in equation (C2). In that case, the constants *K* and *J* should be redefined by the change of base formula.

To obtain the extinction coefficients, least-square fittings were performed. Specifically, equation (C2) is rewritten as

$$\log_{10}\left(\frac{e_{\mathrm{out}}}{e_{\mathrm{in}}}\right)=-J_{\mathrm{OD}}\cdot D=-J_{D}\cdot \mathrm{OD},$$

where *J*_OD_ is defined by *J*_OD_ := *J* × OD and *J*_D_ is defined by *J*_D_ := *J* × *D*. Then, *J*_OD_ and *J*_D_ were calculated from the slopes of the fitted lines in figure 5*a*,*b*, respectively. The extinction coefficient *J* was then obtained as *J* = 0.153 from the slope in figure 5*c*, which plots *J*_OD_ and *J*_D_. The value of *J* = 0.153 corresponds to

$$K=\frac{J}{30.1\times 10^{6}}=5.1\times 10^{-9},$$

which is the extinction coefficient of ACCB1808 in the unit of ml cm^−1^ cell^−1^. The assessed extinction coefficient *K* = 5.1 × 10^−9^ ml cm^−1^ cell^−1^ was used for the prediction of the MR model in §3.2 and the full model in §4.2, and for the demonstration of cultivation planning in §5.
